# Detection of Borreliae in Archived Sera from Patients with Clinically Suspect Lyme Disease

**DOI:** 10.3390/ijms15034284

**Published:** 2014-03-11

**Authors:** Sin Hang Lee, Jessica S. Vigliotti, Veronica S. Vigliotti, William Jones, David M. Shearer

**Affiliations:** 1 Department of Pathology, Milford Hospital, 300 Seaside Ave., Milford, CT 06460, USA; E-Mails: jessvigs@yahoo.com (J.S.V.); ronnie313vig@yahoo.com (V.S.V.); will.jones@milfordhospital.org (W.J.); 2 Therapeutic Research Foundation, Old Saybrook, CT 06475, USA; E-Mail: dshearer@tr-f.org

**Keywords:** DNA sequencing, same-nested PCR, 16S rDNA, Lyme borreliosis, novel borrelia, Lyme disease, *Borrelia burgdorferi*, *Borrelia miyamotoi*, 2-tier serology

## Abstract

The diagnoses of Lyme disease based on clinical manifestations, serological findings and detection of infectious agents often contradict each other. We tested 52 blind-coded serum samples, including 20 pre-treatment and 12 post-treatment sera from clinically suspect Lyme disease patients, for the presence of residual Lyme disease infectious agents, using nested PCR amplification of a signature segment of the borrelial 16S ribosomal RNA gene for detection and direct DNA sequencing of the PCR amplicon for molecular validation. These archived sera were split from the samples drawn for the 2-tier serology tests performed by a CDC-approved laboratory, and are used as reference materials for evaluating new diagnostic reagents. Of the 12 post-treatment serum samples, we found DNA evidence of a novel borrelia of uncertain significance in one, which was also positive for the 2-tier serology test. The rest of the post-treatment sera and all 20 control sera were PCR-negative. Of the 20 pre-treatment sera from clinically suspect early Lyme disease patients, we found *Borrelia miyamotoi* in one which was 2-tier serology-negative, and a *Borrelia burgdorferi* in two—one negative and one positive for 2-tier serology. We conclude that a sensitive and reliable DNA-based test is needed to support the diagnosis of Lyme disease and Lyme disease-like borreliosis.

## 1. Introduction

In North America, Lyme disease is most often caused by *Borrelia burgdorferi* sensu stricto [1]. According to the guidelines of the Centers for Disease Control and Prevention (CDC), its diagnosis is primarily based on symptoms, physical findings (e.g., rash), and the possibility of exposure to infected ticks; laboratory testing is helpful if used correctly and performed with validated methods [2]. However, the clinical manifestations of Lyme disease are highly variable and often not easily distinguished from those caused by other illnesses in clinical practice [3]. The characteristic *erythema migrans* (EM) rash may not display a classic bull’s-eye (ring-within-a-ring) appearance, a fact that may be underappreciated. Some studies noted uncharacteristic variants of EM in 25%–30% of cases. One study reported the rash to be uniformly red in 60% of cases. Other atypical variants of EM are a blue-red appearance and, occasionally, a vesicular central region [4]. As a result, it is not easy to find a group of patients with a definitive diagnosis of “Lyme disease” as the gold standard for comparative studies to further develop new reliable diagnostic tests.

Although initially described as a joint disease [5], Lyme disease is actually a systemic infection presenting with bacteremia at some point during the course of the disease [6–9]. Due to the slow growth rate of the borrelial bacteria in artificial media and the paucity of the microbes in the patients’ blood, the commonly used blood culture is not a useful laboratory test for diagnosing *B. burgdorferi* spirochetemia.

The recommended laboratory test for the diagnosis of Lyme disease is the demonstration of two of the three bands of 24 kDa (OspC), 39 kDa (BmpA), and 41 kDa (Fla) in an IgM immunoblot, in addition to five of the 10 bands of 18 kDa, 21 kDa (OspC), 28 kDa, 30 kDa, 39 kDa (BmpA), 41 kDa (Fla), 45 kDa, 58 kDa (not GroEL), 66 kDa, and 93 kDa in an IgG immunoblot [10]. This test which, also referred to as the “2-tier serology” assay, usually shows the diagnostic immunoblot combination of 2 of the 3 IgM bands and 5 of the 10 IgG bands during convalescence of the infection. It may be negative or non-diagnostic in up to 75% of clinically suspect cases of early Lyme disease [7], and has been the subject of debate when it is relied on for making the diagnosis of Lyme disease for patient management [11,12]. The lack of diagnostic antibodies in the serum of a patient with Lyme disease may have many causes, one of which is the timing of blood testing. In bacterial septicemias, detectable specific antibodies against an infectious agent generally appear at the late stage of bacteremia or after the causative agents have already been cleared from the circulating blood. Since the rate of borrelial clearance from the blood and the immunological responses to the highly complex antigenic compositions of the borrelia species in terms of antibody production may vary from patient to patient, it is reasonable to expect that the blood samples drawn at any one time from the Lyme disease patients may show different antibody characteristics and harbor a variable density of the borrelial bacteria. It is also reasonable to expect that the blood of a Lyme disease patient may be positive for the diagnostic antibody bands, but negative for the causative agent, or *vice versa*, at any time point. However, in the United States, there is no concurrent routine test to detect Lyme disease infectious agents in the blood sample at the time when a 2-tier serology test is performed. Therefore, it is not known how many, if any, of the patients whose 2-tier serology test result was negative or ambiguous, actually harbored an infectious agent of Lyme borreliosis in their blood, namely at a stage of transient spirochetemia.

This study was designed to use 16S ribosomal RNA gene (16S rDNA) sequencing to determine if residual Lyme disease bacteria or bacterial fragments could be detected in the archived serum samples which were drawn from patients with a diagnosis of clinically suspect Lyme disease and had been tested by the 2-tier serology assay. If residual infectious agents can be found in the archived serum samples of Lyme disease patients, a concurrent DNA sequencing-based test for borrelial cells in the whole blood at the time of the serology assay may be warranted as a supplementary diagnostic tool.

Recently, a newly recognized tick-borne human borreliosis—which is caused by *B. miyamotoi*, a spirochete classified in the relapsing fever group, and may present with clinical manifestations mimicking Lyme disease—was reported for the first time in Russia in 2011 [13], and has subsequently been found also in the United States [14,15]. Since the currently used 2-tier serology test is unlikely to be helpful in the diagnosis of *B. miyamotoi* infections [16], the experimental design is also aimed at detection of possible presence of relapsing fever borreliae in these archived serum samples.

## 2. Results and Discussion

### 2.1. Detection of “Lyme Borreliae” in Archived Post-Treatment Sera from Lyme Disease Patients

Under a Material Transfer Agreement with the CDC of the United States, the authors received from the latter agency 32 blind-coded serum samples (100 μL each), including 12 serum samples collected from patients with clinically suspect Lyme disease according to the CDC criteria and had been treated with antibiotics for the infection, and 20 control serum samples collected from patients, two diagnosed with fibromyalgia, two with rheumatoid arthritis, two with multiple sclerosis, two with infectious mononucleosis, two with syphilis and two with severe periodontitis; and from healthy persons, four living in Lyme disease-endemic regions, and four living in non-endemic regions. These CDC samples were serology references, which had been tested by a CDC-approved reference laboratory and were known to contain the standard Lyme disease antibody bands generated by an approved 2-tier serology test kit in the positive samples. They are intended to be used for evaluation of the accuracy of new diagnostic tests for Lyme disease. The samples were shipped to the first author’s laboratory in frozen state over dry ice in a thermo-insulated container by overnight delivery from the Centers for Disease Control and Prevention, Fort Collins, CO, USA.

The serum samples were allowed to thaw at room temperature. The bacteria in the serum were pelleted by centrifugation and the microbial DNA in the pellet was extracted with hot ammonium hydroxide and precipitated in ethanol. The DNA was finally dissolved in 100 μL TE buffer. Two 3 μL aliquots of each DNA extract were used to initiate two same-nested PCRs [17] for each sample in duplicate. The same-nested PCR consisted of a primary PCR and a nested PCR in tandem with one identical pair of M1 and M2 primers for both. The M1 and M2 primers were “genus-specific” primers designed to amplify a highly conserved segment with hypervarable regions of the borrelial16S ribosomal RNA gene DNA (16S rDNA) of all commonly known borrelia species found in the GenBank. The nested PCR products were subjected to agarose gel electrophoresis. The 357–358 bp target amplicon was used as the template for automated two-directional Sanger sequencing with the M1 and M2 nucleotide as the sequencing primer for each direction. A minimum 150-base inter-primer segment of the signature DNA sequence excised from the computer-generated base-calling electropherogram was submitted to the GenBank for BLAST (Basic Local Alignment Search Tool) alignment analysis. A 100% identities (ID) match with a standard sequence on the GenBank report was required for validation of the molecular diagnosis of a borrelial species.

After the testing results were reported to the CDC, the blinded serum samples were decoded. Matching the PCR results with the CDC decoding data (Table 1) showed that among the 32 samples tested only one of the 3 μL duplicate aliquots of the DNA extract from the serum of patient #9 generated a positive M1/M2 PCR amplion.

The #9 patient was diagnosed with “neurologic Lyme disease” and had been treated before the serum sample was drawn. Direct DNA sequencing of the nested PCR amplicon confirmed that the sequence of the amplicon is that of a novel borrelia in the relapsing fever group (see below). Based on previously published nested PCR data [18–20], this result indicated that the number of residual borrelial bacteria or bacterial fragments containing chromosomal DNA in the positive serum sample was <30 per 100 μL.

Since >95% of the *B. burgdorferi* spirochetes suspended in whole blood are usually lost during preparation of the serum samples [21] for serology testing, little efforts have been made to detect infectious agents in the split serum sample when the 2-tier serology test is performed for Lyme disease diagnosis in the United States. In one study reported from Poland, Niścigorska *et al.* found no DNA of *B. burgdorferi* sensu lato in 52 split serum samples of forestry workers although 32 of them showed serology evidence of *B. burgdorferi* senso lato infection [22].

### 2.2. Sequencing of a Highly Conserved Segment of 16S rDNA of a Novel Borrelia

**Figure 1.**
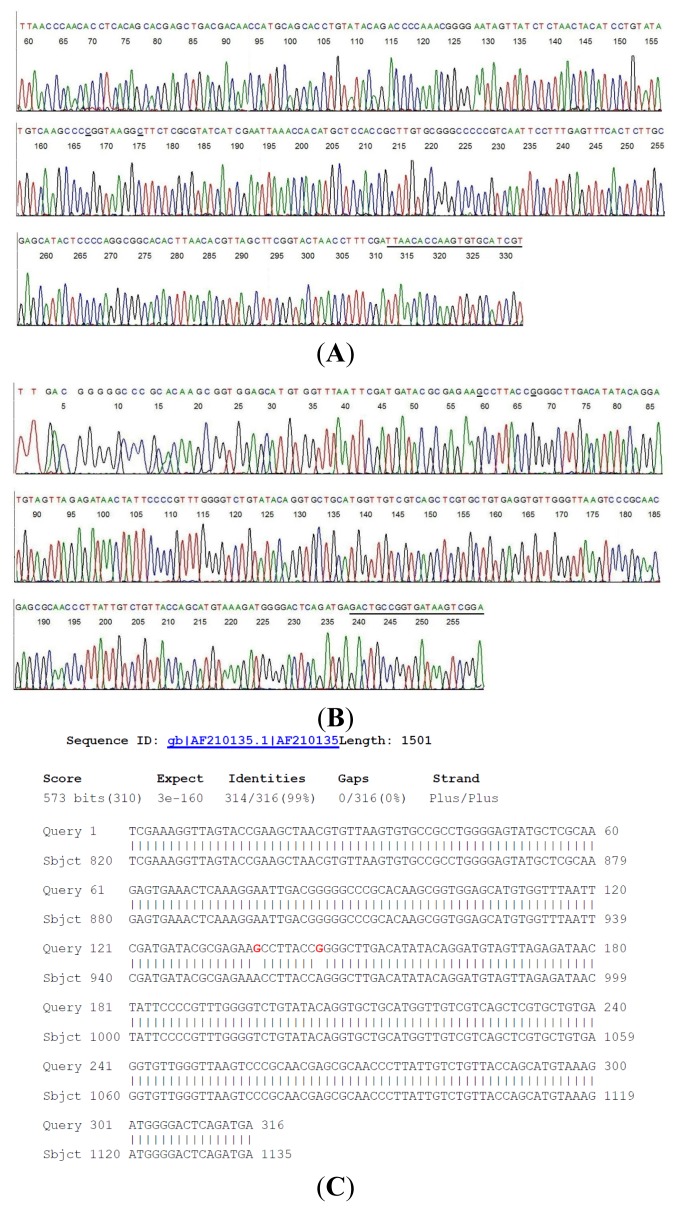
Two-directional sequencing of a highly conserved 358-bp 16S rDNA segment with hypervariable regions of bacterial residues in a human serum sample obtained from a patient diagnosed with “neurologic Lyme disease” (Table 1, #9). (**A**) M2 was the sequencing primer and the M1 primer-binding site is underlined; (**B**) M1 was the sequencing primer and the M2 primer-binding site is underlined; (**C**) *Borrelia coriaceae* isolate CA434 16S ribosomal RNA gene, partial sequence.

A small amount of the 358-bp M1/M2 amplicon from the #9 same-nested PCR tube was transferred directly by a micro-glass rod into 20 μL of automated Sanger reaction mixture [9,18–20] containing the M1 nucleotide or the M2 nucleotide as the sequencing primer. The computer-generated two-directional DNA sequencing base-calling electropherograms, including the primer sites, are shown in Figure 1A,B.

Connecting the two sequences (Figure 1A,B) after all the complementary bases were converted to those for a 5′–3′ reading resulted in a composite inter-primer segment of 316-base 16S rDNA sequence. Submission of this sequence to the GenBank for BLAST alignment generated a returned report. A copy of the report is presented as Figure 1C.

This GenBank report indicated that there is no 100% ID match for the submitted unique DNA sequence with any of the nucleotide sequences stored in the GenBank databases. The closest is a 314/316 bases ID match (Figure 1C) with *B. coriaceae* (AF210135) and a 312/316 bases ID match with *B. hermsii* (CP004146), *B. anserina* (CP005829), *B. parkeri* (CP005851) and *B. turicatae* (AY604974), a group of relapsing fever borreliae. Attempts to further characterize this novel isolate failed due to the limited number of microbes in the serum sample.

**Figure 2.**
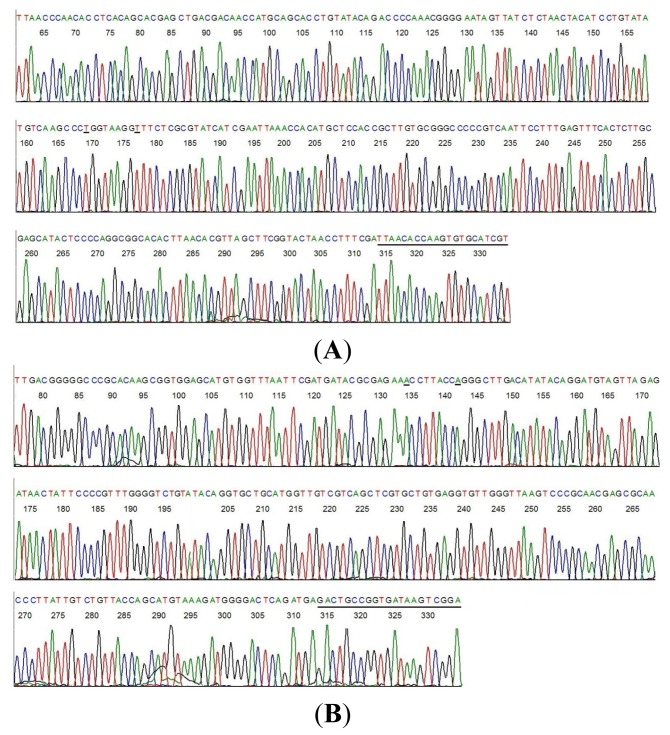
Two-directional sequencing of a highly conserved 358-bp 16S rDNA segment with hypervariable regions of *B. coriaceae*, strain Co53 culture (ATCC 43381). (**A**) M2 was the sequencing primer and the M1 primer-binding site is underlined; (**B**) M1 was the sequencing primer and the M2 primer-binding site is underlined. Note the unambiguous base “A” (underlined) at positions 956 and 964 in Figure 2B, compared to the unambiguous base “G” (underlined) at positions 956 and 964 in Figure 1B. The two complementary bases “T” and “C” in the opposing DNA strand are also underlined in Figures 1A and 2A, respectively.

As shown in the above GenBank report (Figure 1C), the unique 316 bases 16S rDNA segment of the novel borrelia shares a common sequence with *B. coriaceae* with two discordant bases at positions 956 and 964 where the two nucleotide bases “A” have been replaced by two nucleotides “G” (highlighted in red in the above BLAST alignment report). For comparison, the chromosomal DNA of a standard culture of *B. coriaceae*, strain Co53 (ATCC 43381) was extracted and amplified with the M1/M2 same-nested PCR. The two-directional sequencing electropherograms of the corresponding segment of the *B. coriaceae* 16S rDNA are presented in Figure 2.

Based on animal studies, *B. burgdorferi* is highly susceptible to the antibiotics chosen for Lyme disease treatment, and is invariably eliminated from the blood after a course of antibiotic treatment [23]. Therefore, the finding of “Lyme disease bacteria” in the post-treatment serum sample of a patient with neurologic Lyme disease was a surprise. It was more surprising when a novel borrelial 16S rDNA sequence was identified instead of that of *B. burgdorferi* as the residual bacteria in the serum.

PCR amplification of 16S rDNA followed by direct DNA sequencing has been used for construction of the phylogenetic tree of the borrelia species [13,24], thus is highly reliable for molecular diagnosis of borrelial infectious agents in clinical samples. However, the usefulness of 16S rDNA sequencing as a tool in microbial identification is dependent upon two key elements, deposition of complete unambiguous nucleotide sequences into public or private databases and applying the correct “label” to each sequence [25]. A search of the DNA sequence databases of the GenBank confirmed that the nucleotides at positions 956 and 964 of the 16S ribosomal RNA gene of all known borrelia species are always occupied by the adenine base “A”, using the *B. coriaceae* 16S rDNA (GenBank locus #AF210135) as the positions reference. In order to rule out the possibility that the guanine bases “G” at positions 956 and 964 observed in Figure 1B (underlined) were not a result of polymerase-induced mutations which are invariably random, more than 4 same-nested PCRs, each followed by a two-directional sequencing of the PCR amplicon, were performed on the DNA extracts from sample #9 (Table 1) and from the standard *B. coriaceae* culture to confirm the reproducibility of the sequences represented in Figures 1 and 2. All sequences generated from sample #9 and from the standard culture were identical to those illustrated in Figures 1 and 2, respectively.

*B. coriaceae* is a species known to infect the soft tick *Ornithodoros coriaceus*, classified in the relapsing fever group, and not known to be a human pathogen [26]. Whether this novel isolate represents a true mutant of a wild-type *B. coriaceae* or a new borrelia species of the highly heterogeneous relapsing fever group would have to await further studies.

Since the residual bacteria or bacterial remnants of this novel isolate were found in the serum of a patient who had been diagnosed with and treated for “neurologic Lyme disease”, and since the concomitant 2-tier serology test performed on the split sample showed a classic Lyme disease antibody band pattern (Table 1), it is possible that patient #9 might have a mixed infection by a *B. burgdorferi* and a novel relapsing fever borrelia of uncertain significance which was more resistant to the standard regimen of antibiotic treatment than the *B. burgdorferi* co-infectant. Alternatively, this novel bacterium as a sole infectious agent of this patient’s neuroborreliosis might have some common epitopes with the *B. burgdorferi* species, causing a positive Lyme disease 2-tier serology test result in the host’s serum.

### 2.3. Detection of “Lyme Borreliae” in Archived Pre-Treatment Sera from Lyme Disease Patients

Under another Material Transfer Agreement, the authors received from the CDC 20 blind-coded serum samples (100 μL each), including an undisclosed number of serum samples collected from patients who were diagnosed with clinically suspect Lyme disease according to the CDC criteria, but had not been treated with antibiotics for the infection at the time when the serum samples were collected for the 2-tier serology test.

This second batch of 20 serum samples was shipped to the first’s author’s laboratory, and the DNA of the residual bacteria or bacterial fragments in the sera was extracted and subjected to M1/M2 same-nested PCR as described above. The 357–358 bp nested PCR amplicons detected were sequenced for validation. After the testing results were submitted to the CDC, the blinded samples were decoded. Matching the PCR results with the CDC decoding data (Table 2) showed that only 3 samples of the sera taken from 20 patients all with a diagnosis of clinically suspect Lyme disease were positive for infectious agents, including one isolate of *B. miyamotoi* (#39) and two isolates of *B. burgdorferi* sensu lato (#45 and #50). For each PCR-positive serum sample, the 16S rDNA was only amplified successfully in one of the two duplicate PCRs, indicating that the number of borrelial bacteria or bacterial fragments in the 100 μL serum sample was <30.

Since DNA sequencing-based tests for Lyme disease have not been widely used in laboratory medicine in the United States, the direct Sanger sequencing data are presented below as examples for its application in the diagnosis of *B. miyamotoi* and *B. burgdorferi* in clinical materials.

### 2.4. DNA Sequencing-Based Diagnosis of B. miyamotoi and B. burgdorferi

**Figure 3.**
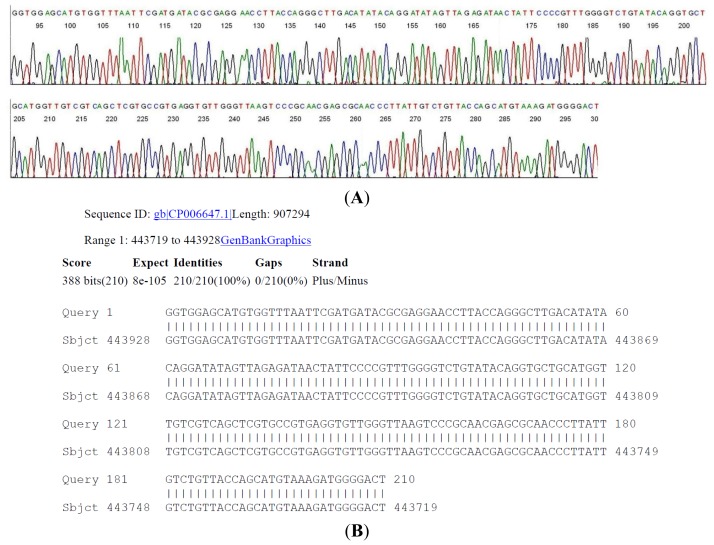
A two-directional sequencing of the 358-bp 16S rDNA same-nested PCR amplicon derived from CDC sample #39 was performed. (**A**) An inter-primer segment of the base-calling electropherogram is illustrated here with a GenBank BLAST report validating the molecular diagnosis of *B. miyamotoi* in the sample; (**B**) Borrelia miyamotoi LB-2001, complete genome.

The 358 bp M1/M2 primer-terminated PCR amplicon derived from serum sample #39 (Table 2) was subjected to two-directional DNA sequencing as described above. A segment of the 16S rDNA signature sequence (Figure 3) was submitted to the GenBank for BLAST sequence alignment analysis, as an example for routinely validating the molecular diagnosis of a *B. miyamotoi* in a clinical laboratory.

It is of interest to note that this patient had a diagnosis of clinically suspect early Lyme disease with *erythema migrans* (EM) and that there were 2 of 3 specific bands in the IgM immunoblot although the 2-tier serology test was interpreted as negative because there were a low EIA value and only 2 bands in the IgG immunoblot (Table 2). A DNA sequencing of an M1/M2 primer-terminated PCR amplicon would have confirmed that patient #39 actually had a *B. miyamotoi* infection with a skin rash, similar to some of the patients reported in the Russian series [13].

**Figure 4.**
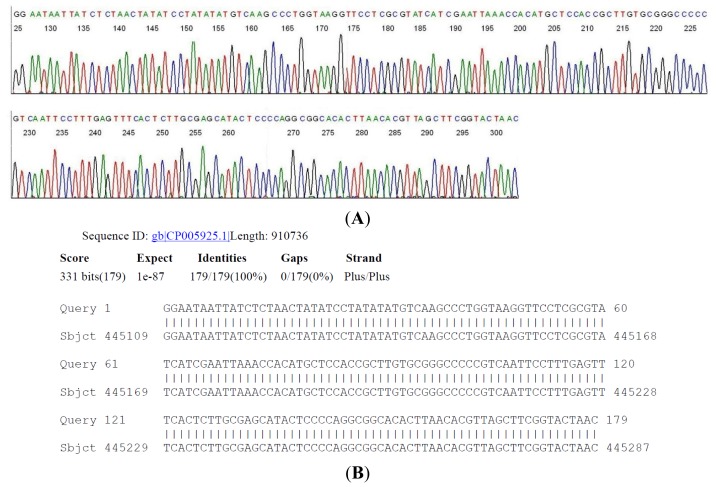
A two-directional sequencing of the 357-bp 16S rDNA same-nested PCR amplicon derived from CDC sample #45 (Table 2) was performed. (**A**) An inter-primer segment of the base-calling electropherogram is illustrated here with a GenBank BLAST report validating the molecular diagnosis of a *B. burgdorferi* in the sample; (**B**) *Borrelia burgdorferi* CA382, complete genome.

The 357 bp M1/M2 primer-terminated PCR amplicon derived from serum sample #45 (Table 2) was subjected to a two-directional DNA sequencing as described above. A segment of the 16S rDNA signature sequence (Figure 4) was submitted to the GenBank for BLAST sequence alignment analysis, as an example for routinely validating the molecular diagnosis of *B. burgdorferi* in a clinical laboratory.

Although patient #45 (Table 2) had a diagnosis of clinically suspect early Lyme disease with EM, the 2-tier serology test was not supportive of the clinical diagnosis due to a low EIA value and only one band observed in the IgG immunoblot with no bands in the IgM immunoblot. For patients similar to case #45, the finding of *B. burgdorferi* 16S rDNA validated by DNA sequencing would be of great value in confirming the clinical diagnosis.

Serum sample #50 was confirmed by PCR and DNA sequencing to be positive for *B. burgdorferi*, similar to serum sample #45. Patient #50 was diagnosed with clinically suspect early Lyme disease with a high EIA value and 2 of 3 specific bands in the IgM immunoblot, but only one IgG band. Although the CDC reference laboratory interpreted the 2-tier serology test results in sample #50 as positive, some practitioners might consider these combinations of bands to be ambiguous or borderline, based on the CDC guidelines which require demonstration of five of the 10 bands in an IgG immunoblot for a positive 2-tier serology test [10]. Patients with a set of 2-tier serology test results like those in case #50 may or may not be diagnosed as having Lyme disease if the clinical presentations are not typical of “Lyme disease”. A reliable test for the infectious agents in EDTA-anticoagulated whole blood samples would be of great help in confirming the clinical diagnosis in these cases. The CDC has not maintained a panel of standard borrelia-positive whole blood samples taken from Lyme disease patients for diagnostic methodology evaluation.

## 3. Experimental Section

### 3.1. Preparation of Borrelial DNA

The CDC serum samples received on dry ice were thawed at room temperature, and centrifuged at ~16,000× *g* for 10 min. After the supernatant was discarded, the pellet was suspended in 100 μL of TE buffer. The DNA in the pellet was extracted in ammonium hydroxide solution, precipitated in ethanol and finally dissolved in 100 μL of TE buffer as previously described [9].

Extraction of the DNA from *B. coriaceae*, strain Co53 (ATCC 43381) in frozen liquid culture purchased from American Type Culture Collection (Manassas, VA, USA) by ammonium hydroxide followed by ethanol precipitation was accomplished according to the procedure previously described for the *B. burgdorferi* culture [9,19].

### 3.2. Same-Nested PCR Amplification

To initiate a primary PCR, 3 μL of the crude DNA extract from the ATCC culture containing 30 copies of borrelial chromosomal DNA, 3 μL of the crude DNA extract from the serum sample or 3 μL water as negative control was mixed with 20 μL of ready-to-use LoTemp^®^ PCR master mix (HiFi DNA Tech, LLC, Trumbull, CT, USA), 1 μL of 10 μmol M1 primer (5′-ACGATGCACACTTGGTGTTAA-3′), and 1 μL of 10 μmol M2 primer (5′-TCCGACTTATCACCGGCAGTC-3′) in a total volume of 25 μL per PCR tube. The thermocycling steps were programmed to 30 cycles at 85 °C for 30 s, 50 °C for 30 s, and 65 °C for 1 min after an initial heating for 10 min at 85 °C, with a final extension at 65 °C for 10 min. A trace of each of the primary PCR products was transferred by a micro-glass rod to another 25 μL complete PCR mixture containing 20 μL of ready-to-use LoTemp^®^ PCR master mix, the M1/M2 primer pair and 3 μL of water for same-nested PCR amplification with identical thermocycling steps as for primary PCR. A band of 357- or 358-bp PCR amplicon visualized on agarose gel electrophoresis was accepted as possible presence of a borrelial 16S rDNA, pending confirmation by direct DNA sequencing of the PCR amplicon.

Cross-contamination is a serious concern in any nested PCR clinical laboratories. However, cross-contamination is not an inherent part of the nested PCR technology. It is rather a function of the clinical laboratory that performs PCR. Negative controls are always negative in this laboratory. Before being allowed to work independently after a course of hands-on practical training, all technologists passed an in-house proficiency test. The latter consists of performing nested PCR on a single batch of 50 simulated samples, about one third (1/3) of which have been randomly spiked with a target DNA and requires a 100% correct result to pass [19,20].

### 3.3. Direct DNA Sequencing

The positive same-nested PCR products were used without purification as the templates for direct automated DNA sequencing [9,19]. The M1 and M2 were the two-directional sequencing primers.

## 4. Conclusions

Testing 52 blind-coded serum samples, including 12 post-treatment and 20 pre-treatment sera from patients with clinically suspect Lyme disease, showed that 1 of 12 post-treatment sera contained residual bacteria of a novel borrelia and 3 of 20 pre-treatment sera were positive for known borrelial bacteria, 1 identified as *B. miyamotoi* and 2 as *B. burgdorferi* based on 16S rDNA sequencing. Only in 1 of the 4 split samples which were PCR-positive, the pattern of the antibody bands of the 2-tier serology test matched the infectious agent detected in the same serum and was confirmed by DNA sequencing. Based on these findings, we conclude that a sensitive and reliable DNA-based test is needed to complement the 2-tier serology assay to support the clinical diagnosis of Lyme disease and Lyme disease-like borreliosis.

## Figures and Tables

**Table 1.** Results of 2-tier serology test and PCR detection of borrelia in split serum samples from post-treatment Lyme disease patients and controls.

Case #	Clinical Diagnosis	Acute/Conv.	EIA Value	IgM WB Bands	IgG WB Bands	2-Tier Interpretation	Borrelia by PCR
1	Early LD-EM	Acute	0.07	-	30	Neg.	Neg.
2	Early LD-EM	Acute	0.39	-	18	Neg.	Neg.
3	Early LD-EM	Acute	0.11	-	45, 41	Neg.	Neg.
4	Early LD-EM	Acute	0.72	41, 23	66, 41	Neg.	Neg.
5	Early LD-EM	Conv.	2.05	41, 23	41, 23	Pos.	Neg.
6	Early LD-EM	Conv.	5.26	39, 23	45, 41, 23, 18	Pos.	Neg.
7	Early LD-EM	Conv.	3.08	41, 23	45, 41, 39, 30, 23	Pos.	Neg.
8	Early LD-EM	Conv.	5.01	41, 39, 23	66, 41, 23	Pos.	Neg.
9	Neurologic Lyme	N	4.24	41, 39, 23	93, 41, 39, 23, 18	Pos.	Novel isolate
10	Neurologic Lyme	N	5.04	41, 23	93, 66, 58, 45, 41, 39, 30, 28, 23, 18	Pos.	Neg.
11	Lyme arthritis	N	5.98	41	93, 66, 58, 45, 41, 39, 23, 18	Pos.	Neg.
12	Lyme arthritis	N	4.47	23	93, 66, 58, 45, 41, 39, 30, 28, 23, 18	Pos.	Neg.
13	Fibromyalgia	N	0.71	-	41	Neg.	Neg.
14	Fibromyalgia	N	0.20	-	-	Neg.	Neg.
15	Rheumatoid arthritis	N	0.07	-	66, 41	Neg.	Neg.
16	Rheumatoid arthritis	N	0.38	-	-	Neg.	Neg.
17	Multiple sclerosis	N	0.48	-	41	Neg.	Neg.
18	Multiple sclerosis	N	0.26	-	41	Neg.	Neg.
19	Mononucleosis	N	3.17	-	-	Neg.	Neg.
20	Mononucleosis	N	0.27	23	-	Neg.	Neg.
21	Syphilis	N	0.32	-	41	Neg.	Neg.
22	Syphilis	N	1.73	23	-	Neg.	Neg.
23	Severe periodontitis	N	0.24	-	41	Neg.	Neg.
24	Severe periodontitis	N	0.22	-	-	Neg.	Neg.
25	Healthy endemic	N	1.29	-	-	Neg.	Neg.
26	Healthy endemic	N	0.74	-	66	Neg.	Neg.
27	Healthy endemic	N	0.60	-	-	Neg.	Neg.
28	Healthy endemic	N	0.19	23	45, 41	Neg.	Neg.
29	Healthy non-endemic	N	0.72	-	66, 41, 39	Neg.	Neg.
30	Healthy non-endemic	N	0.42	23	45, 41	Neg.	Neg.
31	Healthy non-endemic	N	0.49	41	41	Neg.	Neg.
32	Healthy non-endemic	N	1.18	-	-	Neg.	Neg.

The information listed in Table 1 was transferred from the CDC decoding data sheet except the data in the “Borrelia by PCR” column. The 2-tier interpretation was that of the CDC reference laboratory whose EAI cutoff values were: Negative <0.75; equivocal >0.75 to <1.00 and positive >1.00. Conv. = convalescent; EM = *Erythema migrans*; N = not applicable; - = no bands detected.

**Table 2.** Results of 2-tier serology test and PCR detection of borrelia in split serum samples from pre-treatment Lyme Disease patients.

Case #	Clinical Diagnosis	EIA Value	IgM WB Bands	IgG WB Bands	2-Tier Interpretation	Borrelia by PCR
33	Early LD-EM	0.82	-	-	Neg.	Neg.
34	Early LD-EM	4.41	41, 39, 23	41, 23, 18	Pos.	Neg.
35	Early LD-EM	1.29	41	30	Neg.	Neg.
36	Early LD-EM	0.11	23	-	Neg.	Neg.
37	Early LD-EM	0.25	-	23	Neg.	Neg.
38	Early LD-EM	0.49	-	66	Neg.	Neg.
39	Early LD-EM	0.72	41, 23	66, 41	Neg.	*B. miyamotoi*
40	Early LD-EM	0.07	-	30	Neg.	Neg.
41	Early LD-EM	0.25	-	-	Neg.	Neg.
42	Early LD-EM	4.52	41, 39, 23	66, 58, 45, 41, 39, 23, 18	Pos.	Neg.
43	Early LD-EM	5.10	41, 23	66, 58, 45, 41, 39, 23, 18	Pos.	Neg.
44	Early LD-EM	0.21	39, 23	23	Neg.	Neg.
45	Early LD-EM	0.65	-	41	Neg.	*B. burgdorferi*
46	Early LD-EM	0.11	-	45, 41	Neg.	Neg.
47	Early LD-EM	0.80	41, 23	41, 23	Pos.	Neg.
48	Early LD-EM	0.39	-	18	Neg.	Neg.
49	Early LD-EM	2.60	23	66, 41, 23	Neg.	Neg.
50	Early LD-EM	3.76	41, 23	41	Pos.	*B. burgdorferi*
51	Early LD-EM	4.25	41, 39, 23	41	Pos.	Neg.
52	Early LD-EM	1.95	41, 39, 23	58, 41	Pos.	Neg.

The information listed in Table 2 was transferred from the CDC decoding data sheet except the data in the “Borrelia by PCR” column. The 2-tier interpretation was that of the CDC reference laboratory whose EAI cutoff values were: Negative <0.75; equivocal >0.75 to <1.00 and positive >1.00. EM = Erythema migrans; - = no bands detected.
